# MicroRNA-494 inhibits cell proliferation and invasion of chondrosarcoma cells *in vivo* and *in vitro* by directly targeting SOX9

**DOI:** 10.18632/oncotarget.4460

**Published:** 2015-07-01

**Authors:** Jingyuan Li, Lijuan Wang, Zongzhi Liu, Chao Zu, Fanfan Xing, Pei Yang, Yongkang Yang, Xiaoqian Dang, Kunzheng Wang

**Affiliations:** ^1^ Department of Orthopaedics, The Second Affiliated Hospital of Xi'an Jiaotong University, Xi'an, 710004, Shaanxi Province, P.R. China; ^2^ Department of Orthopaedics, Shaanxi Provincial People's Hospital, The Third Affiliated Hospital of Xi'an Jiaotong University, Xi'an, 710068, Shaanxi Province, P.R. China; ^3^ Department of Oncology, The First Affiliated Hospital of Xi'an Jiaotong University, Xi'an, 710061, Shaanxi Province, P.R. China; ^4^ Department of Surgical Oncology, Shaanxi Provincial People's Hospital, The Third Affiliated Hospital of Xi'an Jiaotong University, Xi'an, 710068, Shaanxi Province, P.R. China; ^5^ Department of Clinical Microbiology and Infection Control, The University of Hong Kong - Shenzhen Hospital, Shenzhen, 518053, Guangdong Province, P.R. China; ^6^ Department of Gynecology and Obstetrics, The Second Affiliated Hospital of Shaanxi University of Chinese Medicine, Xianyang, 712000, Shaanxi Province, P.R. China

**Keywords:** miR-494, chondrosarcoma, metastasis, prognosis, SOX9

## Abstract

Accumulating evidence indicates that dysregulation of miRNAs could contribute to tumor growth and metastasis of chondrosarcoma by infuencing cell proliferation and invasion. In the current study, we are interested to examine the role of miRNAs in the carcinogenesis and progression of chondrosarcoma. Here, using comparative miRNA profiling of tissues and cells of chondrosarcoma and cartilage, we identified miR-494 as a commonly downregulated miRNA in the tissues of patients with chondrosarcoma and chondrosarcoma cancer cell line, and upregulation of miR-494 could inhibit proliferation and invasion of chondrosarcoma cancer cells *in vivo* and *in vitro*. Moreover, our data demonstrated that SOX9, the essential regulator of the process of cartilage differentiation, was the direct target and functional mediator of miR-494 in chondrosarcoma cells. And downregulation of SOX9 could also inhibit migration and invasion of chondrosarcoma cells. In the last, we identified low expression of miR-494 was significantly correlated with poor overall survival and prognosis of chondrosarcoma patients. Thus, miR-494 may be a new common therapeutic target and prognosis biomarker for chondrosarcoma.

## INTRODUCTION

Chondrosarcoma is a malignant cartilage forming neoplasm, and the second most frequent primary malignant type of primary bone malignancy after osteosarcoma, accounting for approximately 40% of all primary bone malignancies and presenting a wide spectrum of clinicopathological behaviors [1, 2]. Usually occurring in males aged 10–80 years, and tumors usually appear on scapula, ribs, sternum or pelvis [3, 4]. Conventional chondrosarcoma has been found to be highly resistant to chemotherapeutic regimens and radiotherapy for their extracellular matrix and poor vascularity [5, 6]. More importantly, there is no specific standardized therapy has been developed for this malignancy. Thus, wide local excision remains the important the primary mode of therapy. However, patients with chondrosarcoma usually present relatively poor prognosis after surgical resection because of potent capacity for local invasion and distant metastasis [7]. Therefore, it is an urgent need to identified novel prognostic biomarkers and therapeutic approaches to improve chondrosarcoma clinical management, prevent recurrence and treat inoperable micro-lesions.

MicroRNAs (miRNA) are short non-coding RNAs (approximately 21–24 nucleotides in length), transcribed form non-protein coding genes or introns, which could modulate gene expression at post-transcriptional level and maintain normal cellular functions by binding to complementary sites in their 3′-untranslated region (3′-UTR) [8, 9]. Currently, many miRNAs has been confirmed to play important roles in carcinogenesis and cancer metastasis [10]. Some miRNAs which are overexpressed in cancers act as oncogenes and contribute to the transformed penotypes. These oncogenic miRNAs take effects by suppressing tumor suppressor genes. Other miRNAs which are down-regulated in cancers may act as tumor suppressor miRNAs. They take effects by allowing the expression of oncogenes [11]. Downregulated expression of miRNAs has been reported in multiple types of human cancers and affected multiple steps during the process of metastasis [12]. As far as chondrosarcoma is concerned, accumulating evidence indicates that deregulation of miRNAs can contribute to metastasis of chondrosarcoma by infuencing cell proliferation and invasion, such as miR-145, miR-100 and miR-30a [13–15]. Therefore, it is necessary to identify more miRNAs as potential prognosis predictor and therapeutic target for chondrosarcoma patients.

In the current study, we are interested to examine the role of miRNAs in the carcinogenesis and progression of chondrosarcoma. Here, using comparative miRNA profiling of tissues and cells of chondrosarcoma and cartilage, we identified miR-494 as a commonly downregulated miRNA in the tissues of patients with chondrosarcoma and chondrosarcoma cancer cells, and upregulation of miR-494 could inhibit proliferation and invasion of chondrosarcoma cells *in vitro* and *in vivo*. Moreover, our data demonstrated that SRY-related high mobility group-Box gene 9 (SOX9), the essential regulator of the process of cartilage differentiation, was the direct target gene and functional mediator of miR-494 in chondrosarcoma cells. Downregulation of SOX9 could also inhibit proliferation and invasion of chondrosarcoma cells. In the last, we investigated the clinical relevance of miR-494 in chondrosarcoma. We identified low expression of miR-494 was correlated with poor overall survival and prognosis of chondrosarcoma patients. Thus, miR-494 may be a new common therapeutic target and prognosis biomarker for chondrosarcoma.

## RESULTS

### miR-494 is dwon-regulated in chondrosarcoma tissues and human chondrosarcoma cell line SW1353

**Figure 1:**
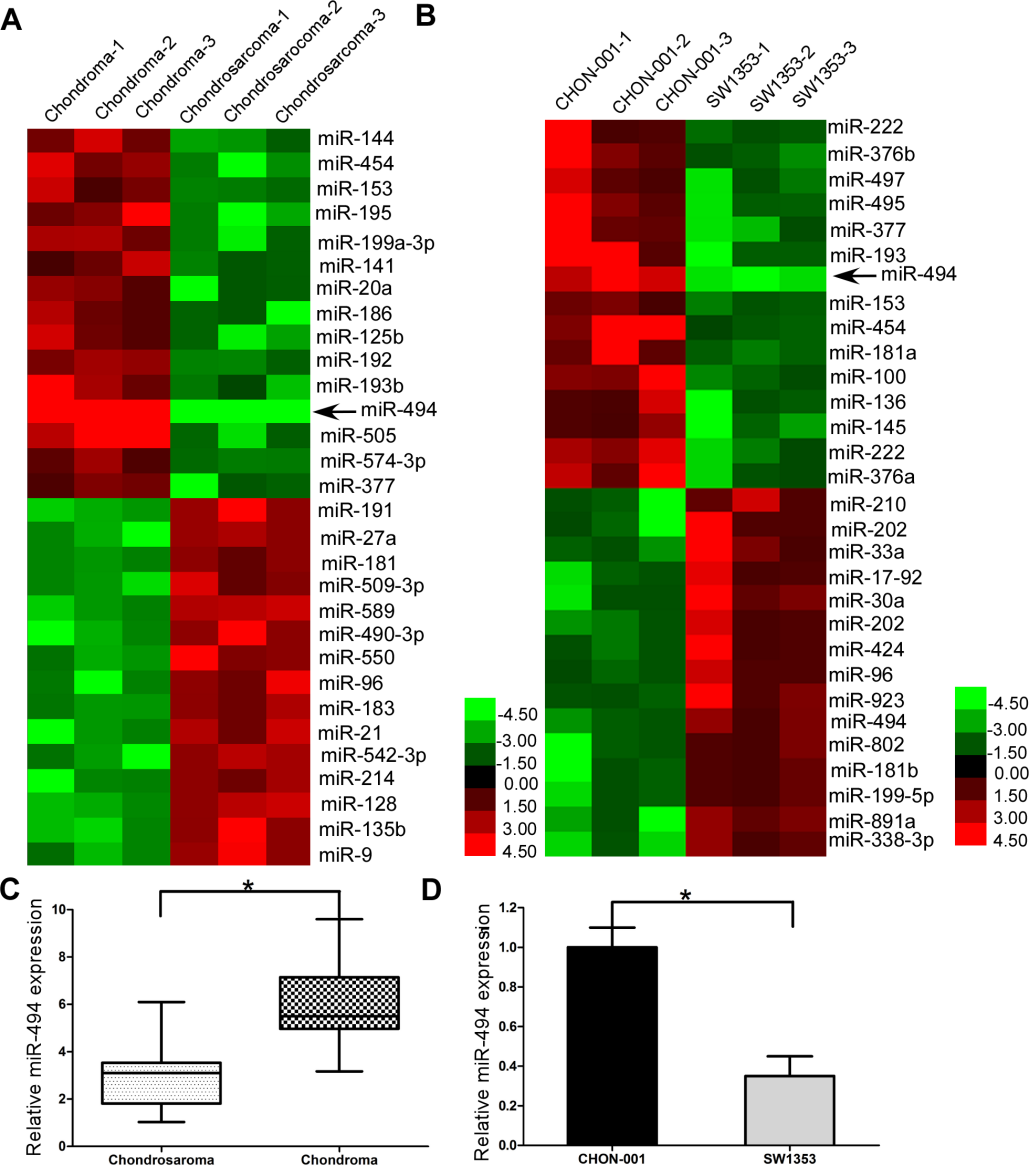
miR-494 is dwon-regulated in chondrosarcoma tissues and human chondrosarcoma cell line SW1353 **A.** miRNA expression between 3 chondrosarcoma tissues and 3 chondroma tissues; **B.** miRNA expression between chondrosarcoma cells and chondrocyte cells; **C.** miR-494 was downregulated in human chondrosarcoma tissues; **D.** miR-494 was downregulated in human chondrosarcoma cell line SW1353. Three independent experiments were performed in duplicate. Data were present as mean ± SD. Two-tailed Student's *t* test was used to analyze the significant differences. *P < 0.05.

To search for the critical miRNAs involved in the carcinogenesis and progression of chondrosarcoma, we extracted total RNA from human chondrosarcoma cells SW1353 and human chondrocyte cells CHON-001, and detected the the expressions of established oncogenic miRNAs and tumor suppressor miRNAs (Supplementary Table 2) by qRT-PCR. We also compared the miRNAs expression between 3 chondrosarcoma tissues and 3 chondroma tissues. Then we presented the expression profiling of miRNAs in tissues and cells of chondrosarcoma and chondroma as heatmaps. From the heatmaps' results, it was identified that miR-494 was significantly downregulated in both human chondrosarcoma tissues and human chondrosarcoma cell line SW1353 (Figure 1A and 1B). Then we further confirmed the significant downregulation of miR-494 in tissues of 71 chondrosarcoma patients (Figure 1C) and chondrosarcoma cell line SW1353 (Figure 1D).

### Effect of miR-494 on cell migration and invasion of chondrosarcoma cells

**Figure 2:**
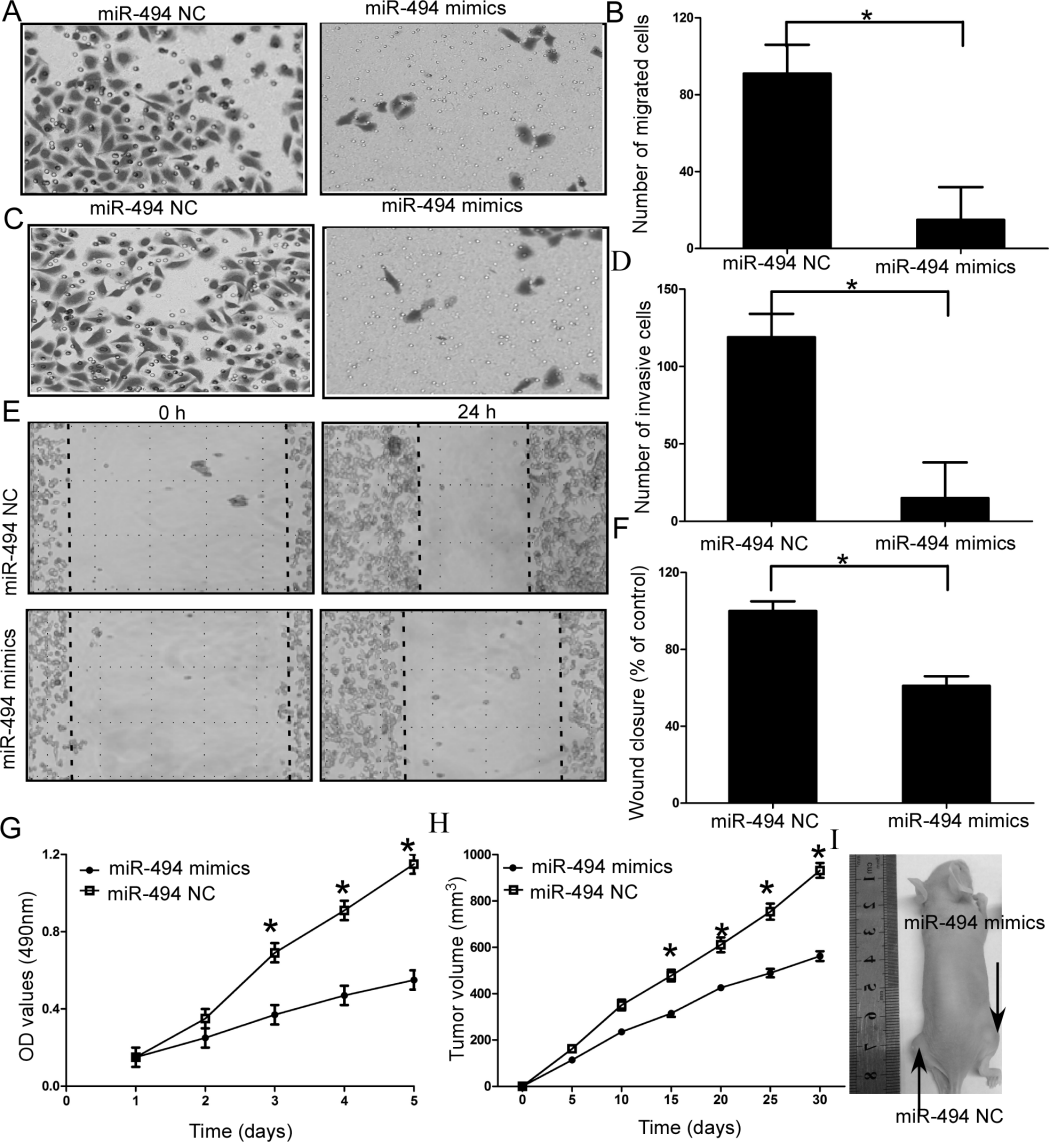
Effects of miR-494 on cell migration and invasion of chondrosarcoma cells **A** and **B.** SW1353 cells transfected with miR-494 mimics significantly decreased the capacity of migration; **C** and **D.** SW1353 cells transfected with miR-494 mimics significantly decreased the capacity of invasion; **E** and **F.** Wound healing assays in the miR-494 mimics transfectants and normal control; **G.** MTT assays of miR-494 mimics and negative control-transfected cells; **H** and **I.** Analysis of tumor growth curves in the SW1353-miR-494 group and SW1353-NC group. Three independent experiments were performed in duplicate. Data were present as mean ± SD. Two-tailed Student's *t* test was used to analyze the significant differences. *P < 0.05.

Given the established suppressor role of miR-494 in cell proliferation and invasion [16, 17], we hypothesized downregulation of miR-494 should account for the maglinant phenotypes of chondrosarcoma cells. To investigate the functional role of miR-494 in the chondrosarcoma cells, we performed gain-of-function experiments by transfecting miR-494 mimics into chondrosarcoma cells. Cell migration and invasion assays results indicated chondrosarcoma cells transfected with miR-494 mimics significantly decreased the capacity of migration and invasion (both *P* < 0.05; Figure 2A–2D, Supplementary Figure 3A and 3B). Wound healing assays also showed significant migration inhibition in the miR-494 mimics transfectants compared to the control in SW1353 cells (*P* < 0.05; Figure 2E–2F).

### Effect of miR-494 on cell proliferation and tumor growth of chondrosarcoma cells *in vitro* and *in vivo*

Then, we investigated the functional role of miR-494 on cell proliferation and tumor growth of chondrosarcoma cells. MTT assays results indicated that overexpression of miR-494 resulted in inhibition of cell proliferation compared to the negative control-transfected cells (NC), (*P* < 0.05; Figure 2G, Supplementary Figure 3C). To further confirm miR-494 functional role on tumor growth *in vivo*, we engineered SW1353 cells to stably upregulate miR-494 expression and performed a tumorigenesis assay in nude mice. The cells were injected into the flanks of nude mice. Tumor sizes were measured every 5 days; after 30 days, the mice were sacrificed and tumors were collected. Analysis of tumor growth curves confirmed that the tumors in the SW1353-miR-494 group grew significantly more slowly than those in SW1353-NC group (*P* < 0.05; Figure 2H and 2I).

### SOX9 is the direct target of miR-494 in chondrosarcoma cells

**Figure 3:**
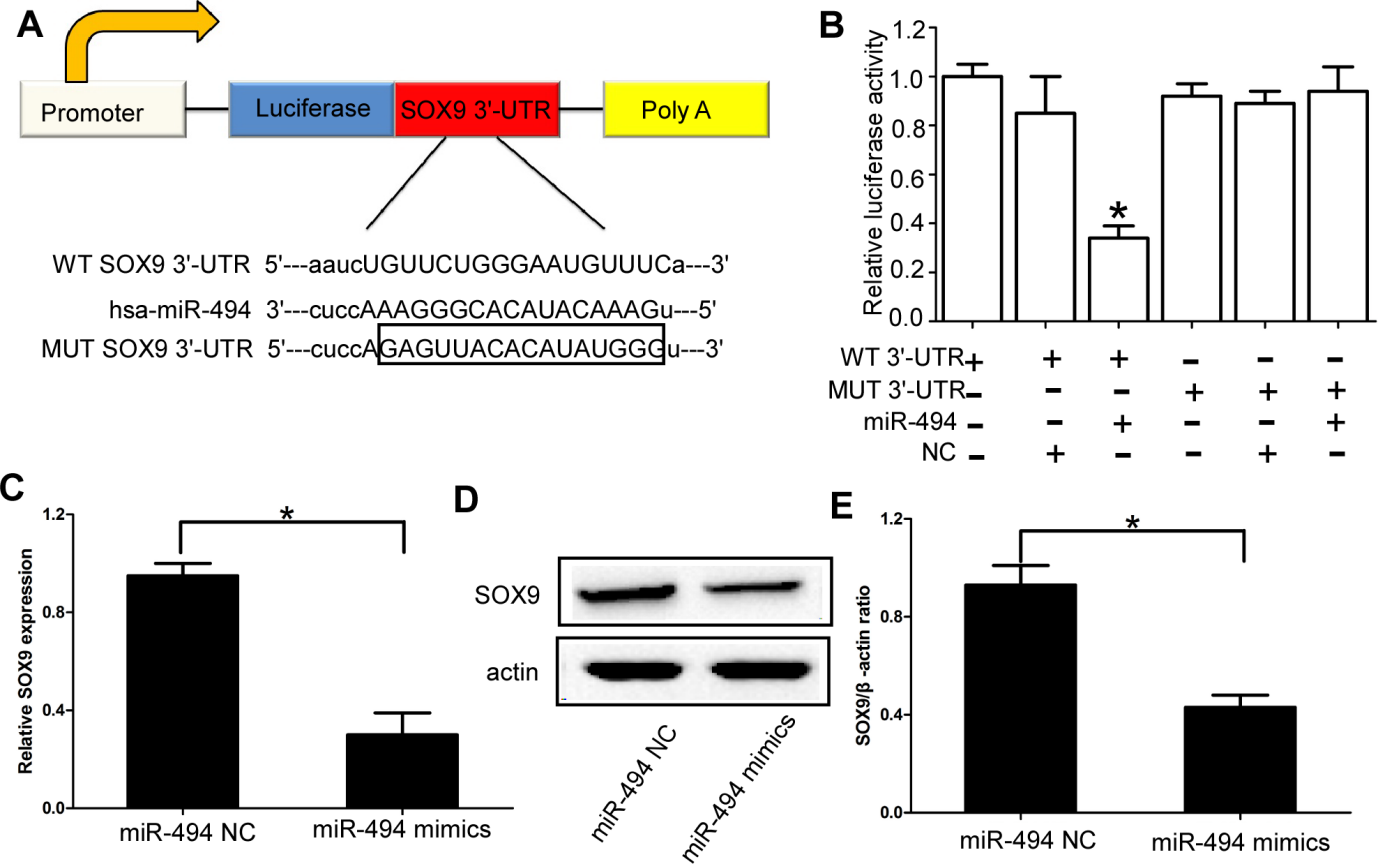
SOX9 is the direct target of miR-494 in chondrosarcoma cells **A.** The target sequence of wild type (WT) SOX9 3′-UTR or mutant (MUT) SOX9 3′-UTR was cloned into a luciferase reporter vector; **B.** The relative luciferase activity in 293T cell after the plasmid with WT or MUT SOX9 3′-UTR co-transfected with miR-494; **C.** Expression of SOX9 at mRNA levels was significantly down-regulated in chondrosarcoma cells transfected with miR-494 mimics; **D** and **E.** Expression of SOX9 at protein levels was significantly down-regulated in chondrosarcoma cells transfected with miR-494 mimics. Three independent experiments were performed in duplicate. Data were present as mean ± SD. Two-tailed Student's *t* test was used to analyze the significant differences. *P < 0.05.

SOX9 has been demonstrated to be highly expressed in chondrosarcoma [18]. More importantly, using TargetScan, miRNAda, and PicTar software, SOX9 was identified as a likely target of miR-494, because it contains a putative miR-494 target site in the 3′-UTR. Thus, we performed luciferase reporter assay to verify whether miR-494 directly binded to the 3′-UTR of SOX9 in chondrosarcoma. The target sequence of wild type (WT) SOX9 3′-UTR or mutant (MUT) SOX9 3′-UTR was cloned into a luciferase reporter vector (Figure 3A). Pre-has-miR-494 or non-functional control miR-NC were co-transfected with the reporter vectors into HEK 293T cells. The miR-494 target sequences and WT SOX9 3′-UTR reduced the relative luciferase activity only when miR-494 was present, but not when the corresponding MUT SOX9 3′-UTR was introduced with miR-494 (Figure 3B). Then we further confirmed SOX9 was the direct target of miR-494 in chondrosarcoma cells by using qRT-PCR and western blot. Our results indicated that the expression of SOX9 at both mRNA and protein levels were significantly down-regulated in chondrosarcoma cells transfected with miR-494 mimics (*P* < 0.05; Figure 3C–3E, Supplementary Figure 2A). We also found the downstream genes of SOX9 were also significantly down-regulated in chondrosarcoma cells transfected with miR-494 mimics (*P* < 0.05; Supplementary Figure 2B).

### Effect of SOX9 on cell migration and invasion of chondrosarcoma cells

**Figure 4:**
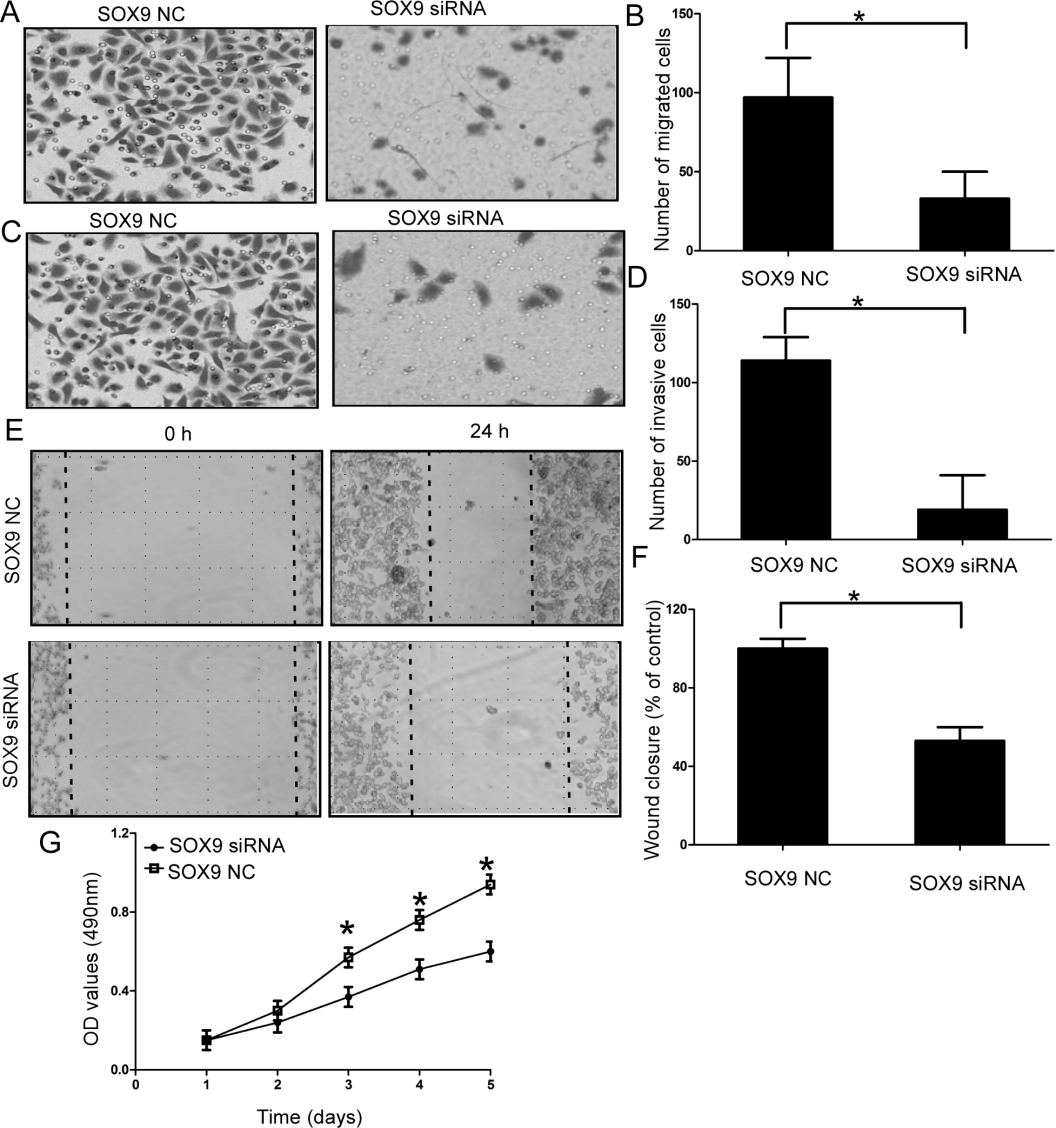
Effect of SOX9 on cell migration and invasion of chondrosarcoma cells **A** and **B.** Silencing SOX9 efficiently inhibited the cell migration of chondrosarcoma cells; **C** and **D.** Silencing SOX9 efficiently inhibited the cell invasion of chondrosarcoma cells; **E** and **F.** Wound healing assays in the SOX9 siRNAs transfectants and normal control; **G.** MTT assays of SOX9 siRNAs and negative control-transfected cells; Three independent experiments were performed in duplicate. Data were present as mean ± SD. Two-tailed Student's *t* test was used to analyze the significant differences. *P < 0.05.

Then we explored the functional role of SOX9 in chondrosarcoma cells, the efficiency of SOX9 siRNA was confirmed by qRT-PCR (Supplementary Figure 1A). Our results showed downregulation of SOX9 efficiently inhibited the cell migration (*P* < 0.05; Figure 4A–4B, Figure 4E–4F, and Supplementary Figure 4A), cell invasion (*P* < 0.05; Figure 4C–4D, Supplementary Figure 4B) and cell proliferation of chondrosarcoma cells (*P* < 0.05; Figure 4G, Supplementary Figure 4C).

### SOX9 is an important functional mediator of miR-494 in chondrosarcoma cells

**Figure 5:**
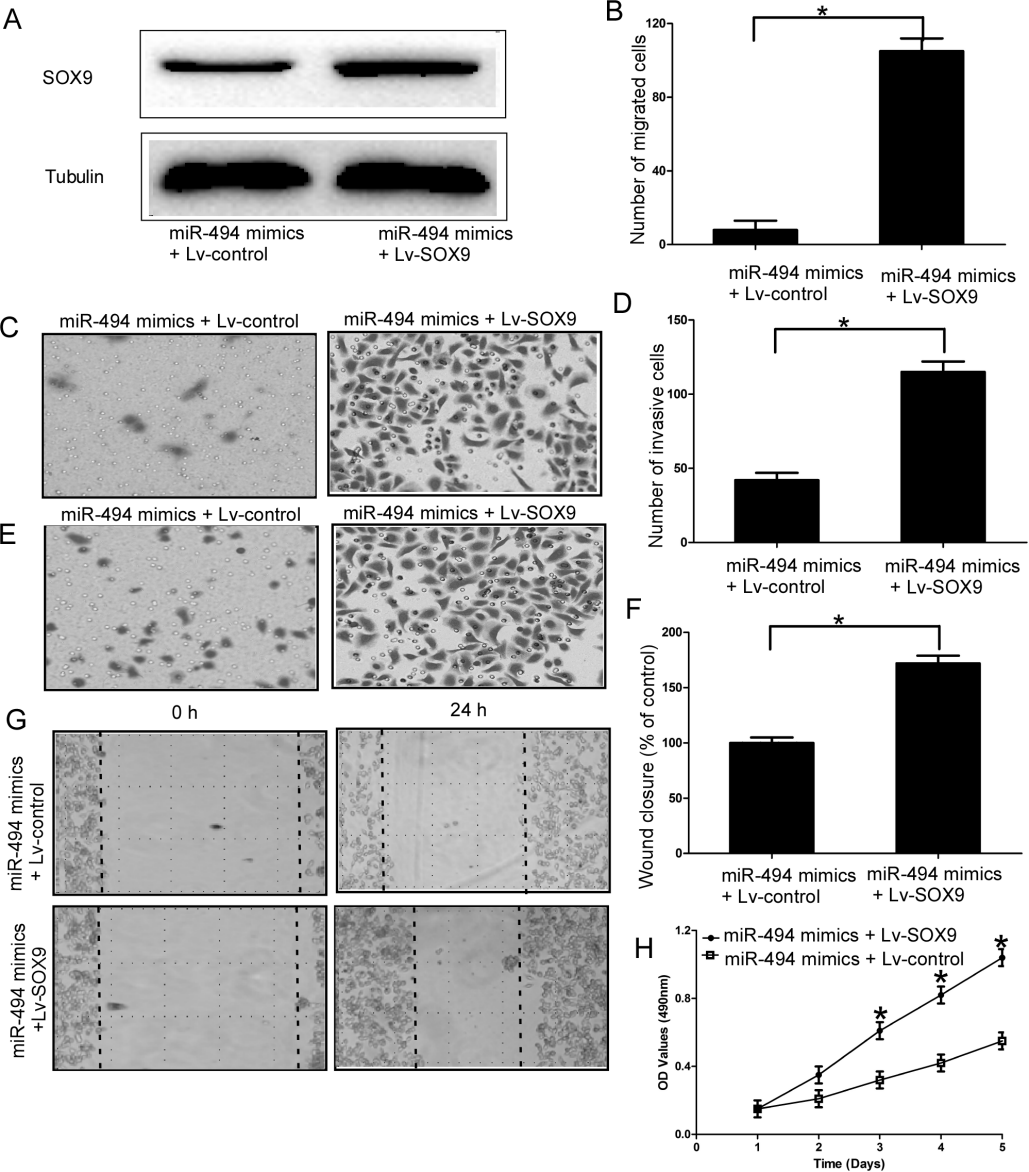
SOX9 is an important functional mediator of miR-494 in chondrosarcoma cells **A.** Protein level of SOX9 in SW1353 cells which trransfected with SOX9 (miR-494 mimics + SOX9 vector group) and miR-494 mimics; **B** and **C.** Exogenous expression of SOX9 rescued the migration capacity induced by overexpression of miR-494 in SW1353 cells; **D** and **E.** Exogenous expression of SOX9 rescued the invasion capacity induced by overexpression of miR-494 in SW1353 cells; **F** and **G.** Wound healing assays in the miR-494 mimics and SOX9 transfectants and miR-494 mimics and EV transfectants; **H.** MTT assays of miR-494 mimics and SOX9 transfectants and miR-494 mimics and EV transfectants. Three independent experiments were performed in duplicate. Data were present as mean ± SD. Two-tailed Student's *t* test was used to analyze the significant differences. *P < 0.05.

To further confirm SOX9 is a functional mediator of miR-494, SOX9 without the 3′-UTR was cloned into a lentivirus vector, and then packaged in 293 T cells by co-transfection with the third generation lentiviral packaging system including pMDGVSVG envelope, pMDLg/pRRE and pRSV-Rev packaging vectors. Then, lv-SOX9 and miR-494 mimics were transfected into chondrosarcoma cells. The upregulation efficiency of lv-SOX9 was determined by qRT-PCR at 48 h after transfection (Supplementary Figure 1B). Our results indicated the protein level of SOX9 was recovered after treatment with SOX9 (miR-494 mimics + lv-SOX9 group) compare to cells transfected with miR-494 (*P* < 0.05; Figure 5A). Furthermore, migration assays (*P* < 0.05; Figure 5B-5C, Supplementary Figure 5A), invasion assays (*P* < 0.05; Figure 5D–5E, Supplementary Figure 5B), wound healing assays (*P* < 0.05; Figure 5F–5G), and MTT assays (*P* < 0.05; Figure 5H, Supplementary Figure 5C) showed that the exogenous expression of SOX9 rescued the phenotype induced by overexpression of miR-494 in chondrosarcoma cells.

### miR-494 inversely correlated with adverse clinicopathological features of chondrosarcoma patients

To determine the clinical significance of miR-494 in chondrosarcoma, we analyzed the association of miR-494 expression with various clinicopathological parameters of chondrosarcoma tissues. we split the 71 patients into two groups based on miR-494 expression levels (low *vs* high) with their mean expression levels as a cutoff point. As shown in Table 1, miR-494 was significantly downregulated in chondrosarcoma patients with metastasis status (*P* = 0.026), and positive lymph node metastasis (*P* = 0.014). However, miR-494 expression was not significantly correlated with gender, age, tumor size.

**Table 1:** Clinical correlation between miR-494 expression and other clinicopathological features in chondrosarcoma

Clinicopathological features	Total No. of patients, *N* =	Low miR-494 group	χ^2^	*p*
**Age (years)**	71 (55.7 ± 10.1)	40	0.191	0.809
**≤ 60**	41	24		
**> 60**	30	16		
**Sex**			0.491	0.592
**Men**	52	28		
**Women**	19	12		
**Tumor size**			0.951	0.348
**≤ 5 cm**	39	24		
**> 5 cm**	32	16		
**Lymph node status**			6.546	0.014*
**Negative**	43	19		
**Positive**	28	21		
**Metastasis status**			5.572	0.026*
**Negative**	44	20		
**Positive**	27	20		

* *P* ≤ 0.05

### miR-494 is inversely correlated with SOX9 expression in the tissues of chondrosarcoma patients

**Figure 6:**
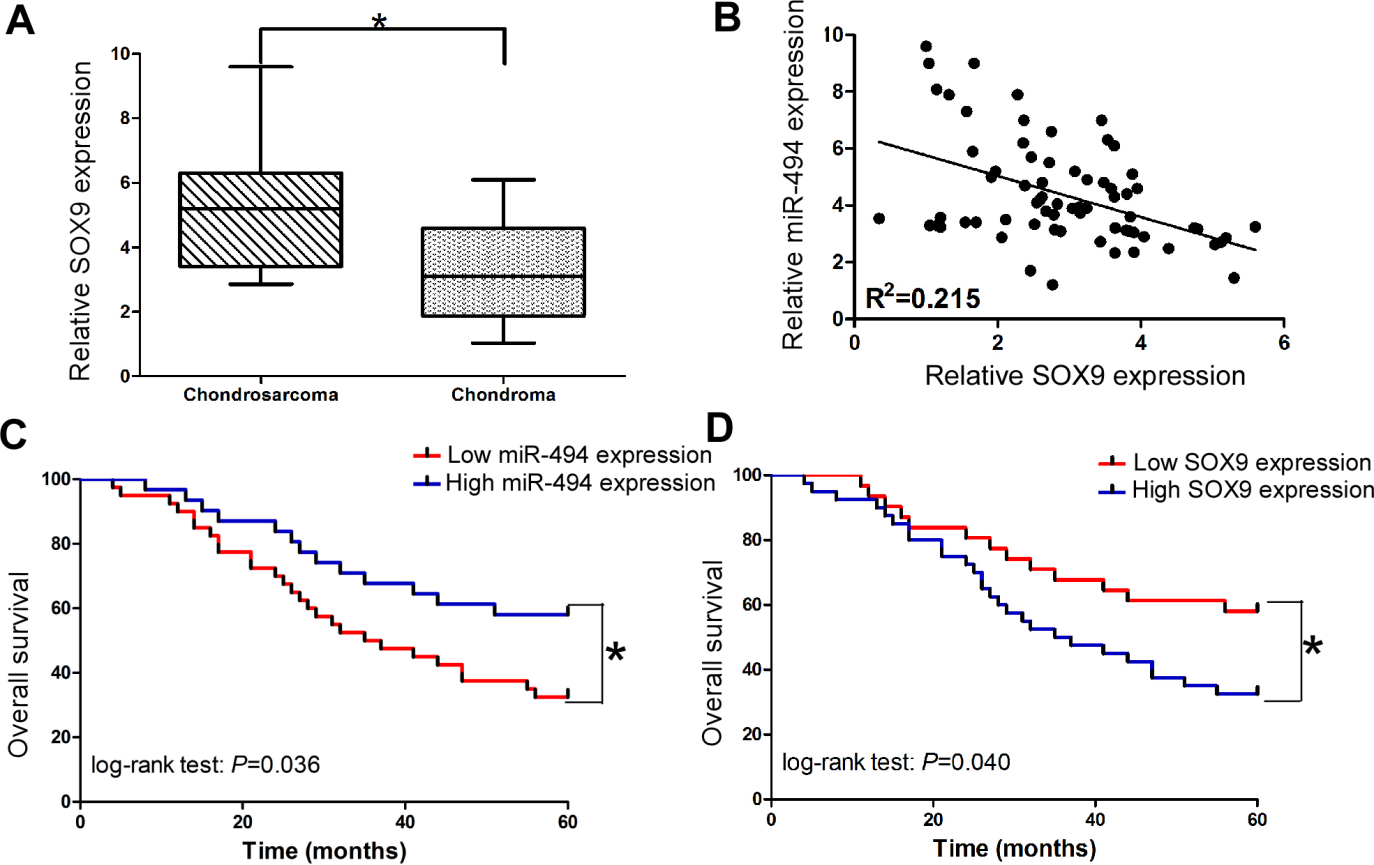
Clinical significance of miR-494 and SOX9 in chondrosarcoma **A.** Relative SOX9 expression in the tissues of chondrosarcoma and chondroma; **B.** The relationships between SOX9 and miR-494 in the tissues of chondrosarcoma; **C.** The relationship between miR-494 expression and overall survival; **D.** The relationship between SOX9 expression and overall survival. Three independent experiments were performed in duplicate. Data were present as mean ± SD. Two-tailed Student's *t* test was used to analyze the significant differences. *P < 0.05.

Then we employed qRT-PCR assay to measure the endogenous expression of SOX9 in the tissues of chondrosarcoma and chondroma. We found SOX9 was significantly upregulated in the tissues of chondrosarcoma (*P* < 0.05; Figure 6A). More importantly, SOX9 expression was inversely correlated with miR-494 in the tissues of chondrosarcoma patients (Figure 6B).

### Low miR-494 expression is correlated with poor overall survival in chondrosarcoma patients

In order to further determine the clinical significance of miR-494 in chondrosarcoma, we also split the 71 patients into two groups based on miR-494 or SOX9 expression levels (low *vs* high) with their mean expression levels as a cutoff point. The Kaplan-Meier analysis revealed that low miR-494 expression was significantly correlated with adversely overall survival in 71 chondrosarcoma patients (Figure 6C; log-rank test: *P* = 0.036). Whereas, the patients with low SOX9 expression tended to obtain better overall survival time than patients with high SOX9 expression (Figure 6D; log-rank test: *P* = 0.040).

### Low miR-494 expression is independent prgnosis predictor for chondrosarcoma

To determine the prognostic value of miR-494 expression in chondrosarcoma, we analyzed the relationships between the miR-494 and clinical outcome. Univariate analysis identified metastasis status, lymph node status, and low expression of miR-494 as poor prognosticators for overall survival (HR = 5.127, CI = 1.314–6.479, *P* = 0.007, Table 2), whereas age, gender and tumor size were not significantly correlated with overall survival. To test whether the prognostic value of low miR-494 expression was independent of other risk factors for poor overall survival, a multivariate analysis was performed using a Cox proportional hazard model. Multivariate analyses including age, gender, tumor size, miR-494 expression, lymph node status, and metastasis status demonstrated that low miR-494 expression was an independent predictor for poor prognosis in chondrosarcoma patients (HR = 3.995, CI = 1.218–5.163, *P* = 0.007, Table 2). Statistically significant results were also obtained for metastasis status, whereas all other parameters were not significant for overall survival.

**Table 2:** Cox regression analysis of prognostic factors for overall survival in chondrosarcoma patients (*n* = 71)

	Univariate			Multivariate		
	HR	95% CI	*P* Value	HR	95%CI	*P* Value
**LowmiR-494 expression**	5.127	(1.314–6.479)	0.012*	3.995	(1.281–5.163)	0.007*
**Age**	1.451	(0.778–1.951)	0.611	1.283	(0.747–1.711)	0.583
**Sex**	1.751	(0.796–1.972)	0.309	1.912	(0.841–2.818)	0.437
**Tumor size**	2.169	(0.861–2.947)	0.411	1.821	(0.519–2.147)	0.377
**Lymph node metastasis**	3.765	(1.248–4.159)	0.032*	2.955	(1.451–3.887)	0.018*
**Metastasis status**	4.361	(1.479–5.287)	0.011*	3.459	(1.291–4.483)	0.009*

95% CI indicates 95% confidence interval

* *P* ≤ 0.05

## DISCUSSION

Unlike Ewing's sarcoma and osetosarcoma, the long-term survival of chondrosarcoma dramatically decreased because of distant metastasis after surgical resection [19, 20]. Thus it is of great significance to identify novel and effective molecular biomarker for chondrosarcoma prognosis and treatment assessment. Here, the main findings of the current study are as following five points. Firstly, we identified miR-494 as a commonly downregulated miRNA in the tissues of chondrosarcoma patients and chondrosarcoma cancer cells by using comparative miRNA profiling of tissues, which is consistent with previous study [21]. Secondly, enforced expression of miR-494 significantly inhibited cell proliferation, cell migration and cell invasion *in vitro*, and inhibited tumor growth *in vivo*. Thirdly, using human chondrosarcoma cell line SW1353 transfected with miR-494 mimics, we validated that SOX9 was downregulated at mRNA and protein level, which was further confirmed by the luciferase reporter assay, suggesting that miR-494 directly binded to SOX9 3′-UTR in chondrosarcoma cells. Fourthly, knockdown of SOX9 also significantly inhibited the cell proliferation, cell migration, and cell invasion of chondrosarcoma cell line *in vitro*, and exogenous expression of SOX9 could rescue the phenotype induced by overexpression of miR-494 in SW1353 cells. Finally, miR-494 was inversely correlated with SOX9 expression in the tissues of chondrosarcoma patients. More importantly, downregulation of miR-494 was significantly correlated with the poor overall survival and prognosis of chondrosarcoma patients. These results suggest that miR-494 could inhibit cell proliferation and invasion of chondrosarcoma cells *in vivo* and *in vitro* by directly decreasing SOX9 expression, and miR-494 is an independent prognosis predictor for chondrosarcoma patients.

Previous reports showed that miR-494 was correlated with several types of human malignant solid tumors, including non small cell lung cancer [22], glioblastoma cells [23], and colorectal cancer [24, 25]. In these types of cancers, miR-494 functions as an oncogenic miRNA, and promotes cancer cell prolifertaion, cell proliferation and invasion via regulation of target genes PTEN, BIM and MMC. But more research demonstrated miR-494 was downregulated in a variety of human malignancies including cholangiocarcinoma [26], gastric cancer [27], esophageal squamous cell carcinoma [16], and ductal adenocarcinoma [28]. In these types of cancers, miR-494 functions its tumor suppressive effects, and inhibites cell proliferation, cell migration, and cell invasion and promoting apoptosis by regulating different oncogenes in different cancer cells, such as VEGF [29], CXCR4 [17], CLPTM1L [16], HOXA10 [30], and c-MYC [27]. However, the functions of miR-494 in chondrosarcoma cells have not yet been studied. In the current study, our results showed miR-494 was downregulated in the tissues of chondrosarcoma patients by using miRNA qRT-PCR assays, and focused on the functions of miR-494 in the proliferation and invasion of chondrosarcoma cells. We demonstrated that miR-494 was functionally involved in suppressing cell proliferation, cell migration, and cell invasion of chondrosarcoma cells by directly binding to 3′-UTR of SOX9. Thus our data indicates miR-494 may play a tumor suppressive role but not a oncogenic role in chondrosarcoma and suppresses the proliferation and invastion of chondrosarcoma cells by decreasing the expression of oncogene SOX9.

SOX9 is developmental gene required for lineage commitment in numerous tissues and organs [31], and is also the essential transcription factor for initial chondrocyte differentiation [32] and cartilage formation [33]. SOX9 is overexpressed in several types of solid tumor and correlated with poor survival and prognosis [34–36]. Upregulation of SOX9 was reported to increase the capacity of cell proliferation, cell migration, and cell invasion in multiple types of cancer [11, 37]. These reports in total indicate the importance to fully delineate the important role that SOX9 played in the carcinogenesis and tumor progression of chondrosarcoma. Previous study has demonstrated that SOX9 was upregulated in chondrosarcoma cells, and miR-145 could inhibit SOX9 expression by directly targeting its 3′-UTR [13]. Considering our results, we think miR-494 and miR-145 may be correlated with each other to inhibit SOX9 expression in chondrosarcoma cells, but the more specific underlying mechanism should be very complicated and need further research. A recent study showed knockdown of SOX9 resulted in apoptposis of human chondrosarcoma cell lines, which indicating SOX9 play an oncogenic role in chondrosarcoma [38]. Consistent with the previous study, we found knockdown of SOX9 could inhibit the cell proliferation, cell migration, and cell invasion of chondrosarcoma cells. More importantly, exogenous expression of SOX9 rescued the phenotype induced by overexpression of miR-494 in SW1353 cells. All these results suggest SOX9 may play an oncogenic role and is a crucial functional mediator of miR-494 in chondrosarcoma cells. More importantly, SOX9 had been proven to participate in Notch 1 induced cell motility, cell invastion and loss of E-cadherin [39], and Notch signaling pathway was found participate in cellular differentiation and proliferation in chondrosarcoma [40]. Thus, future studies are needed to investigate whether SOX9 also participate in Notch 1 induced cell motility and cell invastion. Works is also needed to clarify the mechanisms by which SOX9 activate Notch signaling pathway and upregulated E-cadherin expression in chondrosarcoma.

In conclusion, our findings demonstrated that miR-494 was downregulated in chonrosarcoma cells and tissues of chondrosarcoma patients. Moreover, miR-494 inhibited cell proliferation and invasion of chondrosarcoma cells *in vivo* and *in vitro* by directly binding to the 3′UTR of its functional mediator SOX9. In the last, we identified low expression of miR-494 was correlated with poor overall survival and prognosis of chondrosarcoma patients. Our date indicated miR-494 was a promosing therapeutic target and prognosis biomarker for chondrosarcoma management.

## MATERIALS AND METHODS

### Patients and specimens

This study was approved by the Research Ethics Committee of Xi'an Jiaotong University. Written informed consent was obtained from all of the patients. All specimens were handled and made anonymous according to the ethical and legal standards. 71 cases of conventional chondrosarcoma and corresponding benign chondroma based on accepted clinicopathological and radiological criteria were enrolled in this study. The follow-up information of all participants was updated every 3 months by telephone. The overall survival was defined as the time elapsed from surgery to death. Information regarding the death of patients was ascertained from their family.

### Cell culture

Human chondrosarcoma cell lines SW1353, JJ012 and human chondrocyte cells CHON-001 were obtained from the Cell Bank of the Chinese Academy of Sciences (Shanghai, China), where they were characterized by mycoplasma detection, DNA-Fingerprinting, isozyme detection and cell vitality detection. They were cultured in Dulbecco's modified Eagle's medium (DMEM) (Invitrogen, Carlsbad, CA) mediums supplemented with 10% fetal bovine serum (FBS)(HyClone, Logan, UT) and cultured in a humidified incubator at 37°C in 5%CO2.

### MTT assays

Cells were plated in 96-well plates (0.5 × 104 cells per well) and transfected with NC, miR-494 mimics. 48 h later, 10 μl of MTT reagent (5 mg/ml) was added to each well and cells were incubated at 37°C for another 4 h. The medium was removed, the cells were solubilized in 150 μl of dimethylsulfoxide, and colorimetric analysis was performed (wavelength, 490 nm). One plate was analyzed immediately after the cells adhered (approximately 4 hours after plating), and the remaining plates were analyzed every day for the next 4 consecutive days.

### Migration and invasion assays

Cell migration capacity were measured *in vitro* using transwell migration assays (Millipore, Billerica, MA). For invasion assay, filters were precoated with 30 μL Matrigel basement membrane matrix (BD Biosciences, Bedford, MA) for 30 min. The following procedures were the same for both migration and invasion assays. Chondrosarcoma cells were seeded to Transwell at 1 × 10^4^ cells/well in serum-free medium, and then incubated for 24 h at 37°C in 5% CO2. Cells were fixed in 3.7% formaldehyde for 5 min and stained with 0.05% crystal violet in PBS for 15 min. Cells on the upper side of filters were removed with cotton-tipped swabs; filters were washed with PBS. Cells on the underside of filters were examined and counted under a microscope. Each experiment (performed in triplicate) was repeated at least three times.

### Wound healing assays

For wound-healing migration assay, cells were seeded on 12-well plates at a density of 1 × 10^5^ cells/well in the culture medium; 24 h after seeding, the confluent monolayer of culture was scratched with a fine pipette tip. Migration was visualized by a microscope; rate of wound closure was observed at the time indicated.

### Animal experiments

Six-week-old BALB/c nude mice were purchased from the Model Animal Research Center of Xi'an JiaoTong University (Xi'an, China). All animal procedures were performed in accord to the criteria outlined in the Guide for the Care and Use of Laboratory Animals prepared by the National Academy of Sciences and published by the National Institutes of Health (Bethesda, MD). SW1353 cells were treated accordingly and injected subcutaneously into the hind limb of BALB/c nude mice (5 × 10^6^ cells/mouse). Tumor growth was monitored every 5 days for a total period of 30 days.

### Luciferarse reporter assay

HEK293T cells were seeded in a 96-well plate at 60% confluence. After 24 hours, cells were transfected with 120 ng of miR-494 expression vector or negative control. Cells were transfected with 30 ng of wild type or mutant 3′-UTR of SOX9. The Sox9 3′-UTR was cloned into pMir-Report (Ambion), yielding pMir-Report-Sox9. Mutations were introduced in potential miR-494 binding sites using the QuikChange site-directed mutagenesis Kit (Stratagene). The pRL-SV40 vector (Promega, USA) carrying the Renilla luciferase gene was used as an internal control to normalize the transfection efficiency. Cells were collected 48 hours after transfection, and luciferase activity was measured using a dual luciferase reporter assay system according to the manufacturer's protocol (Promega).

### Rna extraction and quantitative real-time polymerase chain reaction (qrt-pcr)

For miRNA quantification, total miRNA was extracted from the cells using miRNeasy RNA isolation Kit (Qiagen, Valencia, CA, USA), according to the manufacturer's instructions. TaqMan miRNA qRT-PCR (Applied Biosystems, Foster City, CA, USA) were used to detect and quantify miRNA expression. Data were analyzed with 7500 software v.2.0.1 (Applied Biosystems, Foster City, CA, USA), with the automatic Ct setting for adapting baseline and threshold for Ctdetermination. The universal small nuclear RNA U6 (RNU6B) was used as an endogenous control for miRNAs. Each sample was examined in triplicate and the amounts of PCR products produced were nonneoplasticized to RNU6B. The primers of SOX9 and miR-494 was listed in Supplementary Table 1.

### Oligonucleotide transfection

All synthetic miRNAs including negative control (miR-control) and miR-494 were purchased from Shanghai Genechem (Shanghai, China). Silencer Select Negative Control siRNAs (siRNA control), SOX9 siRNA (sense was 5′-GCAGCGACGUCAUCUCCAAdTdT-3′ and anti-sense was 5′-dTdTCGUCGCUGCAGUAGAGGUU-3′) were obtained from Genechem.

### Statistical analysis

Statistical analysis was performed using IBM SPSS statistical software (version 20.0). Survival curves were estimated using the Kaplan-Meier method, and distributions were evaluated by the long-rank test. The differences in characteristics between the 2 groups were examined by the χ2 test or Fisher's exact test. All *P*-values were determined from 2-sided tests, and statistical significance was based on a *P*-value of 0.05.

## SUPPLEMENTARY FIGURES AND TABLES



## Abbreviations

miR-494: microRNA-494
SOX9: SRY-related high mobility group-Box gene 9
WT: wild type
MUT: mutant
3′-UTR: 3′-untranslated region
qRT-PCR: quantitative real-time polymerase chain reaction
NC: negative control-transfected cells

## References

[1] Wunder JS, Nielsen TO, Maki RG, O'Sullivan B, Alman BA (2007). Opportunities for improving the therapeutic ratio for patients with sarcoma. The Lancet Oncology.

[2] Leddy LR, Holmes RE (2014). Chondrosarcoma of bone. Cancer treatment and research.

[3] Barnes R, Catto M (1966). Chondrosarcoma of bone. The Journal of bone and joint surgery British volume.

[4] Pescador D, Blanco J, Corchado C, Jimenez M, Varela G, Borobio G, Gomez MA (2012). Chondrosarcoma of the scapula secondary to radiodermatitis. International journal of surgery case reports.

[5] Kalinski T, Sel S, Kouznetsova I, Ropke M, Roessner A (2009). Heterogeneity of angiogenesis and blood vessel maturation in cartilage tumors. Pathology, research and practice.

[6] Gelderblom H, Hogendoorn PC, Dijkstra SD, van Rijswijk CS, Krol AD, Taminiau AH, Bovee JV (2008). The clinical approach towards chondrosarcoma. The oncologist.

[7] Tsou HK, Chen HT, Hung YH, Chang CH, Li TM, Fong YC, Tang CH (2013). HGF and c-Met interaction promotes migration in human chondrosarcoma cells. PloS one.

[8] Bartel DP (2004). MicroRNAs: genomics, biogenesis, mechanism, and function. Cell.

[9] Kumar MS, Lu J, Mercer KL, Golub TR, Jacks T (2007). Impaired microRNA processing enhances cellular transformation and tumorigenesis. Nature genetics.

[10] Farazi TA, Hoell JI, Morozov P, Tuschl T (2013). MicroRNAs in human cancer. Advances in experimental medicine and biology.

[11] Zhang Y, Guo X, Xiong L, Kong X, Xu Y, Liu C, Zou L, Li Z, Zhao J, Lin N (2012). MicroRNA-101 suppresses SOX9-dependent tumorigenicity and promotes favorable prognosis of human hepatocellular carcinoma. FEBS letters.

[12] Lerner I, Baraz L, Pikarsky E, Meirovitz A, Edovitsky E, Peretz T, Vlodavsky I, Elkin M (2008). Function of heparanase in prostate tumorigenesis: potential for therapy. Clinical cancer research: an official journal of the American Association for Cancer Research.

[13] Mak IW, Singh S, Turcotte R, Ghert M (2015). The epigenetic regulation of SOX9 by miR-145 in human chondrosarcoma. Journal of cellular biochemistry.

[14] Zhu Z, Wang CP, Zhang YF, Nie L (2014). MicroRNA-100 resensitizes resistant chondrosarcoma cells to cisplatin through direct targeting of mTOR. Asian Pacific journal of cancer prevention: APJCP.

[15] Lu N, Lin T, Wang L, Qi M, Liu Z, Dong H, Zhang X, Zhai C, Wang Y, Liu L, Xiang L, Qi L, Han B, Li J (2015). Association of SOX4 regulated by tumor suppressor miR-30a with poor prognosis in low-grade chondrosarcoma. Tumour Biol.

[16] Zhang R, Chen X, Zhang S, Zhang X, Li T, Liu Z, Wang J, Zang W, Wang Y, Du Y, Zhao G (2015). Upregulation of miR-494 Inhibits Cell Growth and Invasion and Induces Cell Apoptosis by Targeting Cleft Lip and Palate Transmembrane 1-Like in Esophageal Squamous Cell Carcinoma. Dig Dis Sci.

[17] Shen PF, Chen XQ, Liao YC, Chen N, Zhou Q, Wei Q, Li X, Wang J, Zeng H (2014). MicroRNA-494-3p targets CXCR4 to suppress the proliferation, invasion, and migration of prostate cancer. The Prostate.

[18] Soderstrom M, Bohling T, Ekfors T, Nelimarkka L, Aro HT, Vuorio E (2002). Molecular profiling of human chondrosarcomas for matrix production and cancer markers. International journal of cancer Journal international du cancer.

[19] Chen PC, Cheng HC, Yang SF, Lin CW, Tang CH (2014). The CCN family proteins: modulators of bone development and novel targets in bone-associated tumors. BioMed research international.

[20] Wu MH, Huang CY, Lin JA, Wang SW, Peng CY, Cheng HC, Tang CH (2014). Endothelin-1 promotes vascular endothelial growth factor-dependent angiogenesis in human chondrosarcoma cells. Oncogene.

[21] Yoshitaka T, Kawai A, Miyaki S, Numoto K, Kikuta K, Ozaki T, Lotz M, Asahara H (2013). Analysis of microRNAs expressions in chondrosarcoma. Journal of orthopaedic research: official publication of the Orthopaedic Research Society.

[22] Bai Y, Sun Y, Peng J, Liao H, Gao H, Guo Y, Guo L (2014). Overexpression of secretagogin inhibits cell apoptosis and induces chemoresistance in small cell lung cancer under the regulation of miR-494. Oncotarget.

[23] Li XT, Wang HZ, Wu ZW, Yang TQ, Zhao ZH, Chen GL, Xie XS, Li B, Wei YX, Huang YL, Zhou YX, Du ZW (2015). miR-494-3p Regulates Cellular Proliferation, Invasion, Migration, and Apoptosis by PTEN/AKT Signaling in Human Glioblastoma Cells. Cell Mol Neurobiol.

[24] Sun HB, Chen X, Ji H, Wu T, Lu HW, Zhang Y, Li H, Li YM (2014). miR494 is an independent prognostic factor and promotes cell migration and invasion in colorectal cancer by directly targeting PTEN. International journal of oncology.

[25] Lim L, Balakrishnan A, Huskey N, Jones KD, Jodari M, Ng R, Song G, Riordan J, Anderton B, Cheung ST, Willenbring H, Dupuy A, Chen X, Brown D, Chang AN, Goga A (2014). MicroRNA-494 within an oncogenic microRNA megacluster regulates G1/S transition in liver tumorigenesis through suppression of mutated in colorectal cancer. Hepatology.

[26] Olaru AV, Ghiaur G, Yamanaka S, Luvsanjav D, An F, Popescu I, Alexandrescu S, Allen S, Pawlik TM, Torbenson M, Georgiades C, Roberts LR, Gores GJ, Ferguson-Smith A, Almeida MI, Calin GA (2011). MicroRNA down-regulated in human cholangiocarcinoma control cell cycle through multiple targets involved in the G1/S checkpoint. Hepatology.

[27] He W, Li Y, Chen X, Lu L, Tang B, Wang Z, Pan Y, Cai S, He Y, Ke Z (2014). miR-494 acts as an anti-oncogene in gastric carcinoma by targeting c-myc. Journal of gastroenterology and hepatology.

[28] Li L, Li Z, Kong X, Xie D, Jia Z, Jiang W, Cui J, Du Y, Wei D, Huang S, Xie K (2014). Down-regulation of microRNA-494 via loss of SMAD4 increases FOXM1 and beta-catenin signaling in pancreatic ductal adenocarcinoma cells. Gastroenterology.

[29] Chen S, Zhao G, Miao H, Tang R, Song Y, Hu Y, Wang Z, Hou Y (2015). MicroRNA-494 inhibits the growth and angiogenesis-regulating potential of mesenchymal stem cells. FEBS letters.

[30] Liborio-Kimura TN, Jung HM, Chan EK (2015). miR-494 represses HOXA10 expression and inhibits cell proliferation in oral cancer. Oral oncology.

[31] Capaccione KM, Hong X, Morgan KM, Liu W, Bishop JM, Liu L, Markert E, Deen M, Minerowicz C, Bertino JR, Allen T, Pine SR (2014). Sox9 mediates Notch1-induced mesenchymal features in lung adenocarcinoma. Oncotarget.

[32] Oh CD, Lu Y, Liang S, Mori-Akiyama Y, Chen D, de Crombrugghe B, Yasuda H (2014). SOX9 regulates multiple genes in chondrocytes, including genes encoding ECM proteins, ECM modification enzymes, receptors, and transporters. PloS one.

[33] Bobick BE, Chen FH, Le AM, Tuan RS (2009). Regulation of the chondrogenic phenotype in culture. Birth defects research Part C, Embryo today: reviews.

[34] Xia S, Feng Z, Qi X, Yin Y, Jin J, Wu Y, Wu H, Feng Y, Tao M (2015). Clinical implication of Sox9 and activated Akt expression in pancreatic ductal adenocarcinoma. Medical oncology.

[35] Grimont A, Pinho AV, Cowley MJ, Augereau C, Mawson A, Giry-Laterriere M, Van den Steen G, Waddell N, Pajic M, Sempoux C, Wu J, Grimmond SM, Biankin AV, Lemaigre FP, Rooman I, Jacquemin P (2014). SOX9 regulates ERBB signalling in pancreatic cancer development. Gut.

[36] Bruun J, Kolberg M, Nesland JM, Svindland A, Nesbakken A, Lothe RA (2014). Prognostic Significance of beta-Catenin, E-Cadherin, and SOX9 in Colorectal Cancer: Results from a Large Population-Representative Series. Frontiers in oncology.

[37] Dynoodt P, Speeckaert R, De Wever O, Chevolet I, Brochez L, Lambert J, Van Gele M (2013). miR-145 overexpression suppresses the migration and invasion of metastatic melanoma cells. International journal of oncology.

[38] Ikegami D, Akiyama H, Suzuki A, Nakamura T, Nakano T, Yoshikawa H, Tsumaki N (2011). Sox9 sustains chondrocyte survival and hypertrophy in part through Pik3ca-Akt pathways. Development.

[39] Capaccione KM1, Hong X, Morgan KM, Liu W, Bishop JM, Liu L, Markert E, Deen M, Minerowicz C, Bertino JR, Allen T, Pine SR2 (2014). Sox9 mediates Notch1-induced mesenchymal features in lung adenocarcinoma. Oncotarget.

[40] Siar CH1, Ha KO, Aung LO, Nakano K, Tsujigiwa H, Nagatsuka H, Ng KH, Kawakami T (2010). Immunolocalization of notch signaling protein molecules in a maxillary chondrosarcoma and its recurrent tumor. Eur J Med Res.

