# Supplementary material for: MicroRNA-494 inhibits cell proliferation and invasion of chondrosarcoma cells in vivo and in vitro by directly targeting SOX9
Source: Oncotarget. 2015 Jul 1;6(28):26216–29. doi: 10.18632/oncotarget.4460 (PMC4694896; doi:10.18632/oncotarget.4460)
Supplement: Supplementary file 1 [file oncotarget-06-26216-s001.pdf]

SUPPLEMENTARY FIGURES AND TABLES

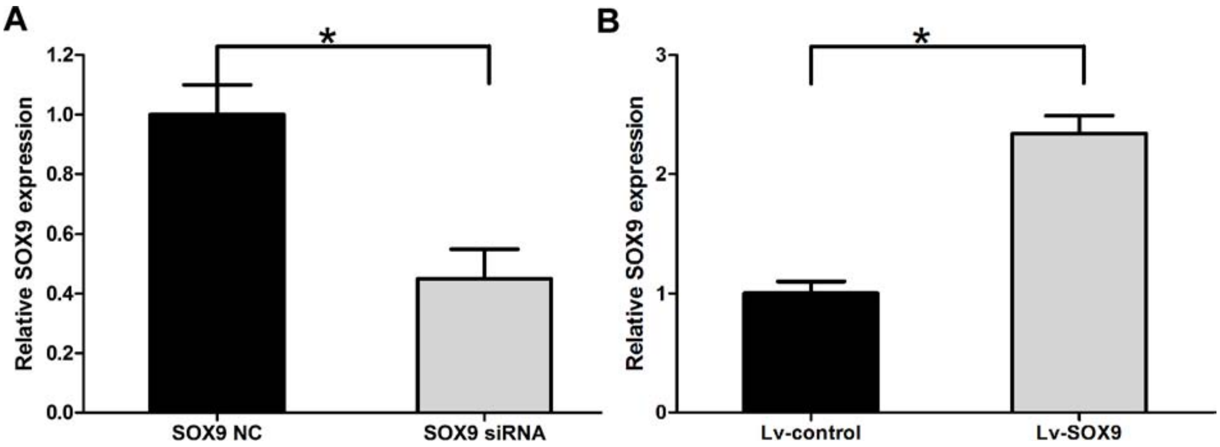

Supplementary Figure S1: Efficiency of SOX9 siRNA and Lv-SOX9 in SW1353 at 48 h. A. Knonckdown efficiency of SOX9 siRNA in SW1353 cells. **B.** Upregulation efficiency of Lv-SOX9 in SW1353 cells.

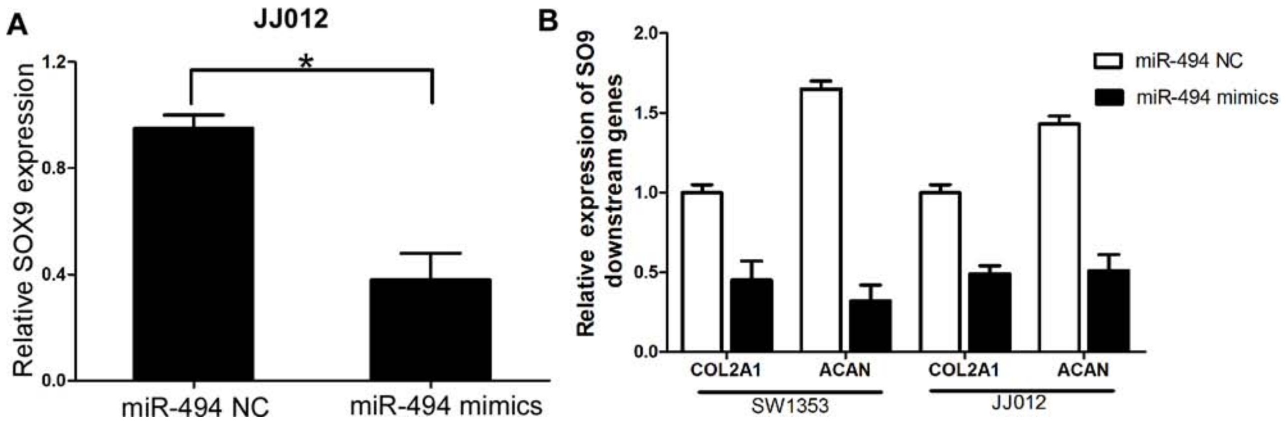

Supplementary Figure S2: SOX9 is the direct target of miR-494 in chondrosarcoma cell line JJ012. A. Expression of SOX9 at mRNA levels was significantly down-regulated in chondrosarcoma cell line JJ012 transfected with pre-miR-494; **B.** Expression of SOX9 downstream genes were significantly down-regulated in chondrosarcoma cell line SW1353 and JJ012 transfected with miR-494 mimics. Three independent experiments were performed in duplicate. Data were present as mean ± SD. Two-tailed Student's *t* test was used to analyze the significant differences. *$P < 0.05$.

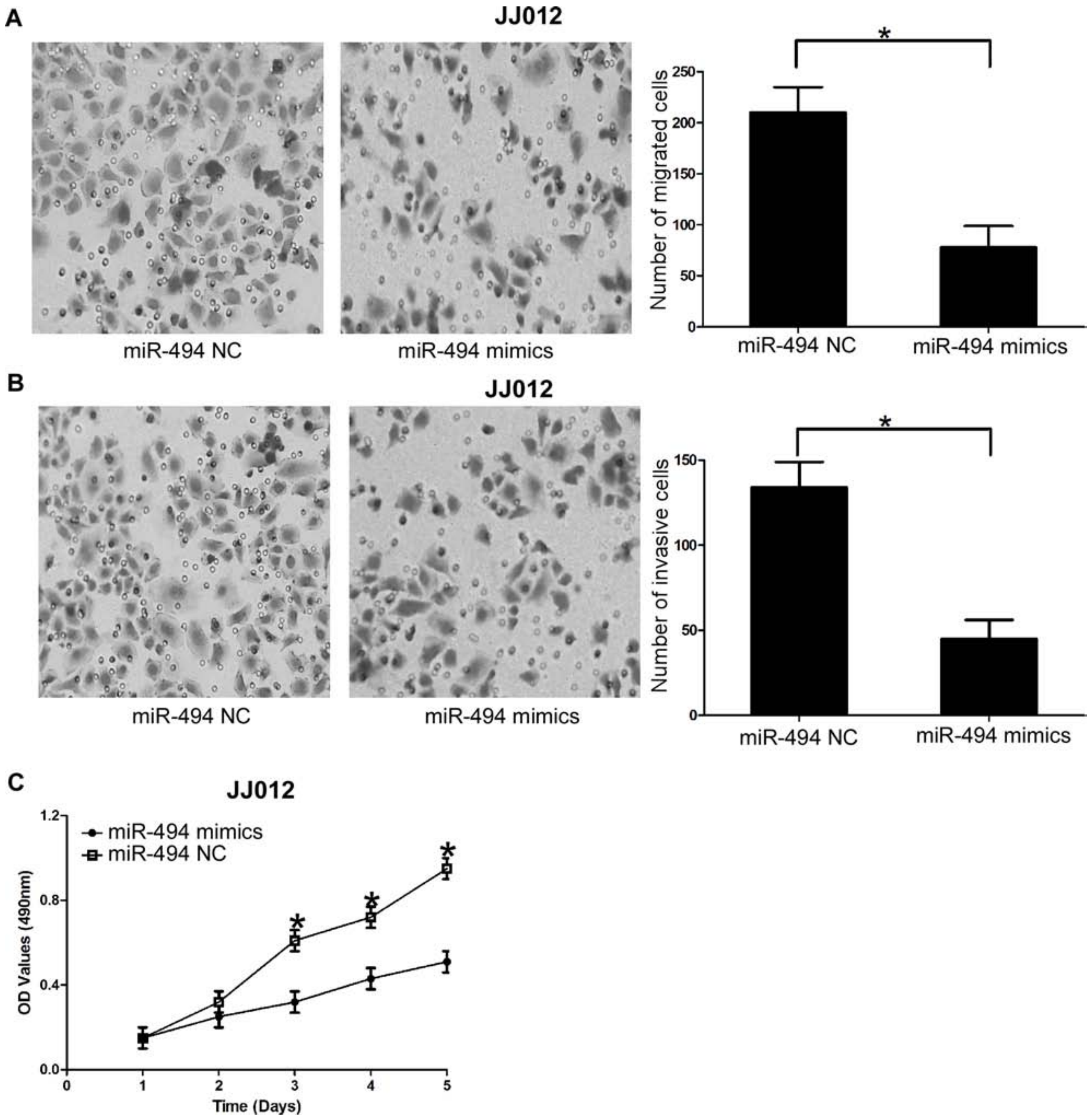

Supplementary Figure S3: Effects of miR-494 on cell migration, cell invasion and cell proliferation of chondrosarcoma cell line JJ012. A. JJ012 cells transfected with miR-494 mimics significantly decreased the capacity of migration; **B.** JJ012 cells transfected with miR-494 mimics significantly decreased the capacity of invasion; **C.** MTT assays of miR-494 mimics and negative control-transfected JJ012 cells.

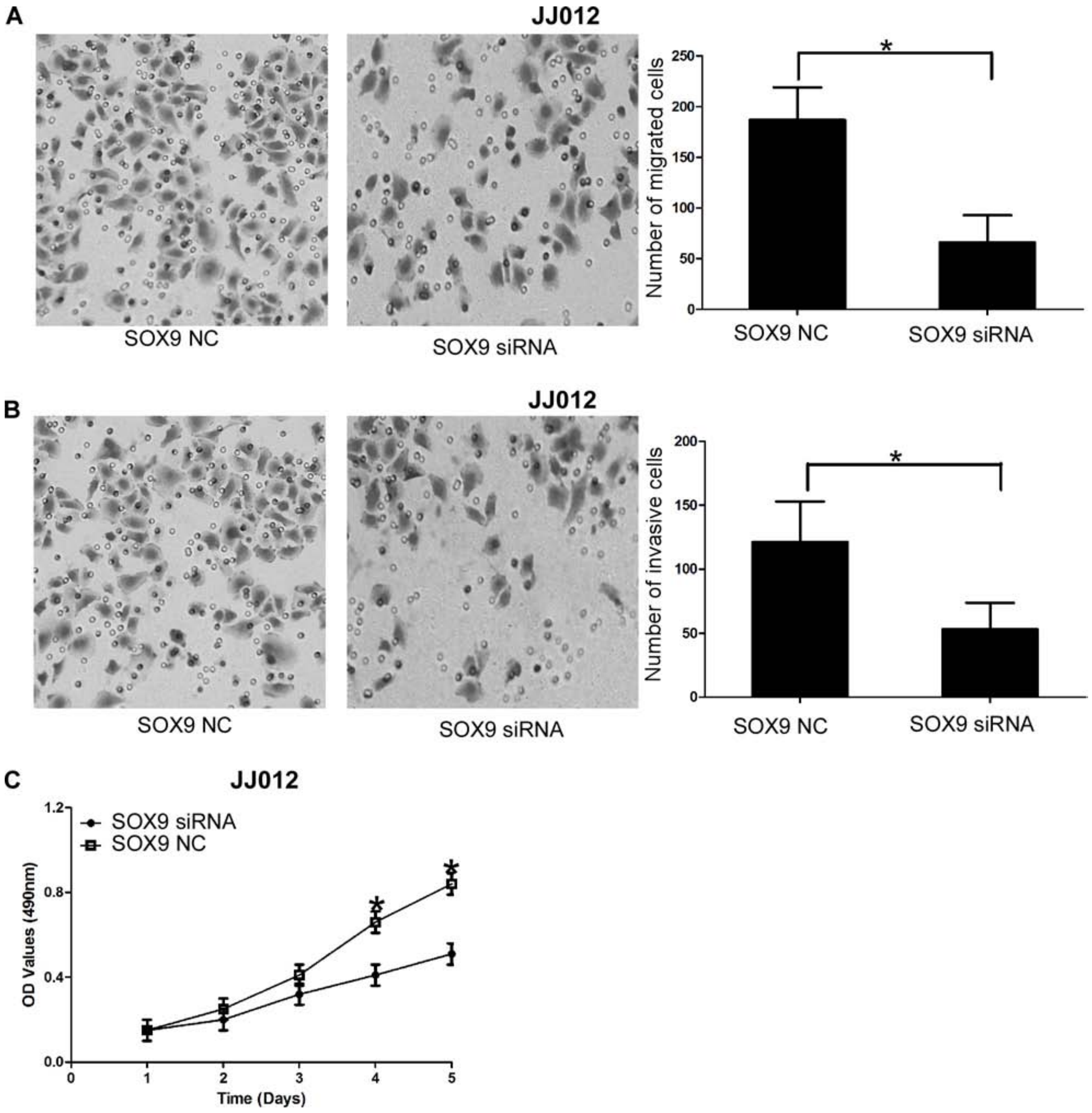

Supplementary Figure S4: Effects of SOX9 on cell migration, cell invasion and cell proliferation of chondrosarcoma cell line JJ012. A. JJ012 cells transfected with SOX9 siRNAs significantly decreased the capacity of migration; **B.** JJ012 cells transfected with SOX9 siRNAs significantly decreased the capacity of invasion; **C.** MTT assays of SOX9 siRNAs and negative control-transfected JJ012 cells.

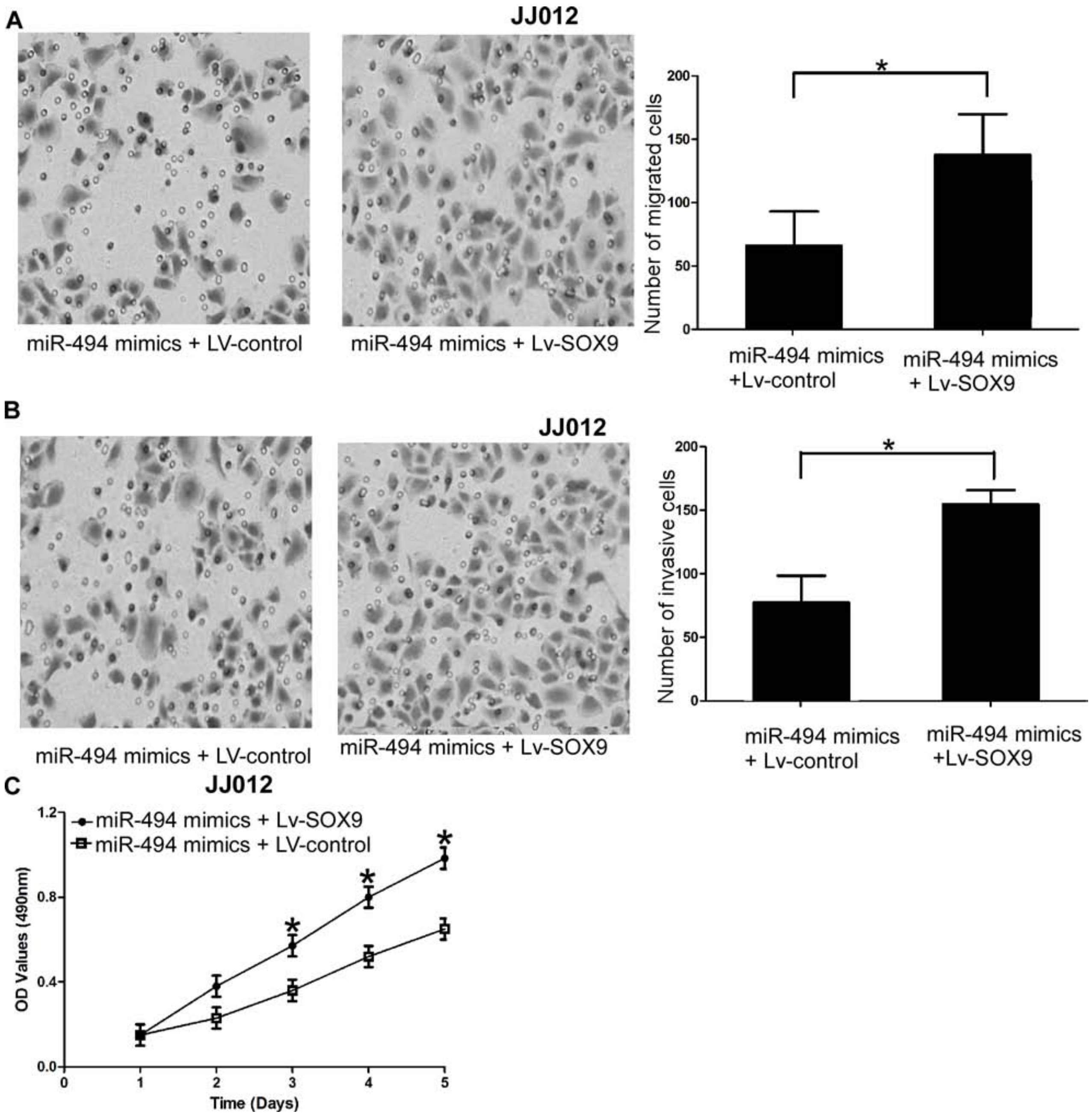

Supplementary Figure S5: SOX9 is an important functional mediator of miR-494 in chondrosarcoma cell line JJ012. A. Exogenous expression of SOX9 rescued the migration capacity induced by overexpression of miR-494 in JJ012 cells; **B.** Exogenous expression of SOX9 rescued the invasion capacity induced by overexpression of miR-494 in JJ012 cells; **C.** MTT assays of miR-494 mimics and SOX9 transfectants and miR-494 mimics and EV transfectants in chondrosarcoma cell line JJ012. Three independent experiments were performed in duplicate. Data were present as mean ± SD. Two-tailed Student's t test was used to analyze the significant differences. *$P < 0.05$.

Supplementary Table S1. Primers used for qRT-PCR.

Gene name	Forward primer 5′-3′	Reverse primer 5′-3′
SOX9	GAG GAA GTC GGT GAA GAA CG	GGA GTG CAC CTC GCT CAT
miR-494	UGAAACAUACACGGGAAACCUC	

Supplementary Table S2. Established oncogenic or tumor suppressor miRNAs detected by qRT-PCR in chondrosarcoma cells and tissues.

Oncogenic miRNAs	Tumor suppressor miRNAs
MicroRNA-191	microRNA-199a-3p
MicroRNA -27a	MicroRNA-144
MicroRNA -181a	MicroRNA -454
MicroRNA -509–3p	MicroRNA-153
MicroRNA -589	MicroRNA-195
MicroRNA -490–3p	MicroRNA-145
MicroRNA -550	MicroRNA-141
MicroRNA -96	MicroRNA-20a
MicroRNA -183	MicroRNA-186
MicroRNA-21	MicroRNA-125b
MicroRNA-542–3p	MicroRNA-192
MicroRNA-214	MicroRNA-193b
MicroRNA-128	MicroRNA-494
MicroRNA-135b	MicroRNA-505
MicroRNA-9	MicroRNA-574–3p
MicroRNA-25	MicroRNA-377
MicroRNA-196a	MicroRNA- 495
MicroRNA-196b	MicroRNA-497
MicroRNA-133a	MicroRNA-376c
MicroRNA-181a	MicroRNA-222
MicroRNA-17–92	MicroRNA-335
MicroRNA-210	MicroRNA-337–5p
MicroRNA-202	MicroRNA-376a
MicroRNA-33a	MicroRNA-376a*
MicroRNA-802	MicroRNA-376b
MicroRNA-181b	MicroRNA-100
MicroRNA-199b-5p	MicroRNA-518b
MicroRNA-891a	MicroRNA-10b
MicroRNA-338–3p	MicroRNA-23b
MicroRNA-494	MicroRNA-24–1*
MicroRNA-30a	MicroRNA-497
MicroRNA-34b	MicroRNA-505
MicroRNA-99b	MicroRNA-27b
MicroRNA-202	MicroRNA-376a*
MicroRNA-125–3p	MicroRNA-193
MicroRNA-218	MicroRNA-21

(*Continued*)

Oncogenic miRNAs	Tumor suppressor miRNAs
MicroRNA-301a	MicroRNA-22
MicroRNA-424	MicroRNA-185
MicroRNA-450a	MicroRNA-195
MicroRNA-483–5p	MicroRNA-376c
MicroRNA-503	MicroRNA-660
MicroRNA-652	MicroRNA-152
MicroRNA-923	MicroRNA-221
MicroRNA-149	MicroRNA-98
